# Biosynthesis of silver nanoparticles with antimicrobial and anticancer properties using two novel yeasts

**DOI:** 10.1038/s41598-021-95262-6

**Published:** 2021-08-04

**Authors:** Xin Liu, Jia-Le Chen, Wen-Yu Yang, Yu-Cheng Qian, Jing-Yu Pan, Chen-Nianci Zhu, Li Liu, Wen-Bin Ou, Hong-Xin Zhao, Dian-Peng Zhang

**Affiliations:** 1 Zhejiang Province Key Laboratory of Plant Secondary Metabolism and Regulation, College of Life Sciences and Medicine, Zhejiang Sci-Tech University, Hangzhou, 310018 China; 2 Institute of Plant and Environmental Protection, Beijing Academy of Agricultural and Forestry Sciences, Beijing, 100097 China; 3 Present Address: Biomass Energy Engineering Research Centre, School of Agriculture and Biology, Shanghai Jiao Tong University, 800 Dongchuan Road, Shanghai, 200240 China

**Keywords:** Biological techniques, Biotechnology

## Abstract

AgNPs are nanomaterials with many potential biomedical applications. In this study, the two novel yeast strains HX-YS and LPP-12Y capable of producing biological silver nanoparticles were isolated. Sequencing of ribosomal DNA-ITS fragments, as well as partial D1/D2 regions of 26S rDNA indicated that the strains are related to species from the genus *Metschnikowia*. The BioAgNPs produced by HX-YS and LPP-12Y at pH 5.0–6.0 and 26 °C ranged in size from 50 to 500 nm. The antibacterial activities of yeast BioAgNPs against five pathogenic bacteria were determined. The highest antibacterial effect was observed on *P. aeruginosa*, with additional obvious effects on *E. coli* ATCC8099 and *S. aureus* ATCC10231. Additionally, the BioAgNPs showed antiproliferative effects on lung cancer cell lines H1975 and A579, with low toxicity in Beas 2B normal lung cells. Therefore, the AgNPs biosynthesized by HX-YS and LPP-12Y may have potential applications in the treatment of bacterial infections and cancer.

## Introduction

Silver nanoparticles (AgNPs) with a size ranging from 1 to 100 nm have notable physical, chemical, and biological characteristics, which are dependent on their size, shape, composition, crystallinity, and structure. AgNPs with unique physical and chemical properties have been used as catalysts^1^, bio-pharmaceuticals^2^, waste treatment reagents^3^, fertilizer additives^4^, and biomedical materials^5,6^.

Antibiotics, which were widely used since 1945 have become an important defense against bacterial infection. However, the long-term use of antibiotics results in the increasing resistance of bacteria, which would in turn lead to larger doses of antibiotics thus further increase the resistance of bacteria^7,8^. This has forced researchers to look for new antimicrobial agents to replace antibiotics. Silver has an excellent bacteriostatic and disinfectant effect. When the size of silver particles is reduced to the nanometer level, its bacteriostatic effect becomes more prominent, so nano-silver has a great advantage for antibacterial applications^9^.

Cancer metastasis is one of the leading causes of death in cancer patients^10^. The treatment of cancer mainly includes traditional surgery, radiotherapy, and chemotherapy, which often do great harm to patients and often do not produce a fully curative effect^11^. Recently, a number of researchers have developed novel treatments with the help of nanotechnology. Among them, nano silver was found to disrupt the telomerase stability mechanism thus inhibiting tumor cell growth^12^.

There are three main methods for synthesizing AgNPs—physical synthesis, chemical synthesis and biosynthesis. In early studies, silver nanoparticles were mainly prepared by physical methods. Solid dispersion is used to convert metallic silver (Ag) into nanoparticles directly. The principle of the physical method is clear and the operation is simple. The particle size of elemental silver is reduced to the nanometer range by electromagnetic, mechanical and laser methods, so that the silver particles have the physical and chemical properties of nanomaterials^13^. However, with the application of physically produced silver nanomaterials, their shortcomings became increasingly clear, such as difficulty in size control, poor uniformity of particle size, and high cost^14^. Chemical synthesis methods rely on the control of chemical reaction conditions, the silver particle size can reach as low as a few nanometers, but the chemical synthesis reaction conditions are harsh, chemical waste causes environmental pollution, and the product also often contains a lot of impurities. By contrast, biosynthesis makes use of biological extracts or microbial fermentation broth, which is mixed with a solution containing silver ions, followed by the separation and purification of nano silver^15–17^. The BioAgNPs obtained by biosynthesis not only have the common physical properties of ordinary AgNPs, but also have special biological characteristics, such as antibacterial activity and biocompatibility. Microbial synthesis is one of the most popular methods for the mass production of AgNPs due to its lower cost and higher output. In recent years, increasing numbers of studies have focused on BioAgNP biosynthesis methods using microorganisms, mainly including actinomycetes^18^, plant leaf extracts, and fungi^19^. It was reported that the synthetically active substances of fungi and actinomycetes are mainly extracellular, and BioAgNPs can be obtained by simply incubating the microorganisms with silver nitrate. The *Actinomyces* sp. strain NH28, which has the ability to synthesize BioAgNPs, was isolated from acidic soil^18^. BioAgNPs with high antibacterial activity were synthesized by *Aspergillus terreus* HA1N and *Penicillium expansum* HA2N^20^. Therefore, a number of biomolecules can act as reducing and protecting agents in the synthesis of BioAgNPs, including products derived from fungi, bacteria, plant extracts, and algae^21^.

In this study, BioAgNPs were synthesized using two novel yeast strains isolated from saline-alkali areas of Qinghai province. The BioAgNPs were examined by UV–vis spectroscopy and scanning electron microscopy (SEM) analysis. Furthermore, the antibacterial efficacy of the BioAgNPs against five pathogenic bacteria was evaluated, and their cytotoxicity against normal lung cells was compared with lung cancer cells. The two novel yeast strains offer a new green approach to synthesize BioAgNPs with potential anticancer and antibacterial applications.

## Materials and methods

### Strains, culture media and reagents

**Figure 1:**
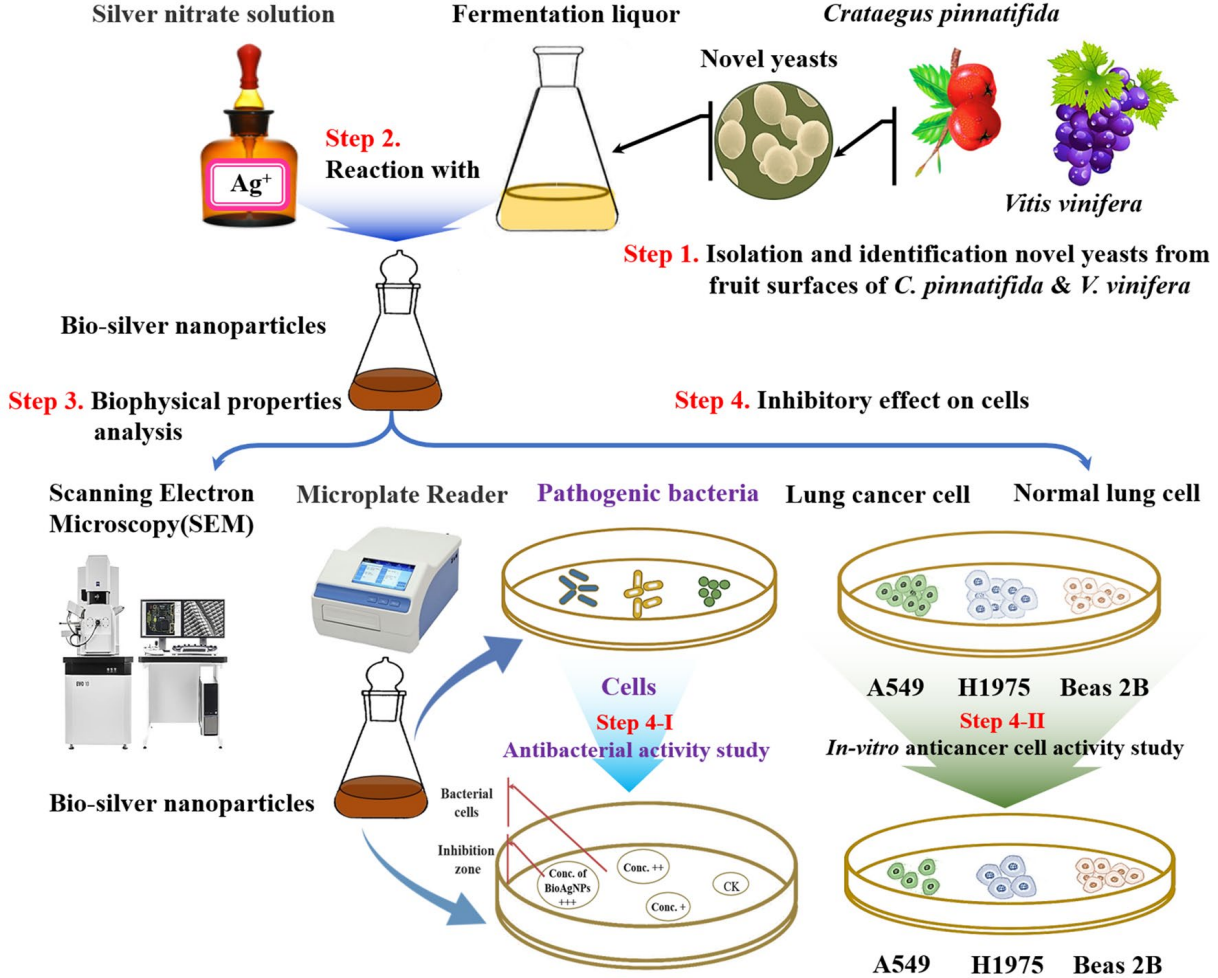
Schematic illustration of experimental process in this study.

The two yeast strains HX-YS and LPP-12Y were isolated from the surfaces of *Crataegus pinnatifida* and *Vitis vinifera* respectively in Qinghai province of China(Fig. 1 Step 1). Five pathogenic indicator strains, including Gram negative (*Escherichia coli* ATCC8099, *Pseudomonas aeruginosa*) and Gram positive bacteria (*Staphylococcus aureus* ATCC6538, *Bacillus subtilis* ATCC6051, *Monilia albicans* ATCC10231) were used to test the antibacterial activity of BioAgNPs synthesized by HX-YS and LPP-12Y.

Potato Dextrose Broth (PDB ) medium containing 26 g potato dextrose powder diluted to 1000 mL with deionized water and adjusted to pH 7.0, was used to culture the yeast strains. LB (Luria–Bertani) medium containing 10 g tryptone, 10 g sodium chloride, 5 g yeast extract, was used to culture the pathogenic bacteria. Biolog Universal Yeast Agar (BUY Agar ) plates containing 60 g BUA agar medium, pH 5.4 ± 0.4 were used to analyze the carbon utilization patterns of the yeast strains.

Human lung cancer cell lines A549 and H1975, as well as the normal human lung epithelial cell line Beas 2B were maintained in our laboratory and regularly screened for mycoplasma contamination using a Mycoplasma Staining Assay Kit (Beyotime Biotechnology, Shanghai). Cells were cultured in standard medium prepared by combining 500 mL of RPMI 1640 with 60 mL of Fetal Bovine Serum (FBS) and adding 1% pen-strep and 1% L-glutamine. The 3-(4,5-dimethylthiazol-2-yl)-2,5-diphenyltetrazolium bromide (MTT) assay was used to assess cell viability in the cytotoxicity tests.

The yeast genomic DNA extraction kit was purchased from Takara (Takara Bio Inc., Dalian, China). Taq DNA Polymerase and High-fidelity DNA Polymerase were purchased from Thermo Scientific (Waltham, MA, USA). The DNA gel extraction kit was purchased from GenScript (Scotch Plains, NJ, USA). Unless noted otherwise, all other chemicals including silver nitrate, pure ethanol, nitric acid, and sodium hydroxide, were of analytical grade and were purchased from Sigma-Aldrich (St. Louis, MO, USA).

### DNA extraction and gene sequencing

DNA extraction was conducted using a yeast genomic DNA extraction kit (Takara Bio Inc., Dalian, China) to identify the yeast strains. Polymerase chain reaction (PCR) with two universal primers, ITS1 (5′-TCCGTAGGTGAACCTGCG G-3′) and ITS4 (5′-TCCTCCGCTTATTGATATGC-3′), was used to amplify ribosomal DNA-ITS fragments of each strain. The universal primers NL1 (5′-GCAT ATCAATAAGCGGAGGAAAAG-3′) and NL4 (5′-GGTCCGTGTTTCAAGACG G-3′) were used to amplify the 26S rDNA D1/D2 regions. The PCR temperature program encompassed initial denaturation for 5 min at 94 °C followed by 30 cycles of 50 s at 94 °C, 45 s at 50–60 °C and 1 min at 72 °C, with a final extension for 10 min at 72 °C and final stored at 4 °C indefinitely. A sequence-similarity search was conducted using BLAST^22^ search in the GenBank database (https://www.ncbi.nlm.nih.gov/).

The morphology of the cells was observed by transmission electron microscopy (TEM-1230; JEOL, Japan), using exponentially growing cells incubated on PDA agar media in Petri dish at 28 °C for 36 h. For transmission electron microscopy, cells were fixed with 2.0% (v/v) glutaraldehyde and 2.0% (v/v) osmium tetroxide. After a 1.5-h fixation in 2.5% glutaraldehyde, the sample was washed three times with 0.1 M phosphate-buffered saline (PBS), followed by two rinses with distilled water, then dehydrated in ethanol solutions of 50%, 70%, 80%, 90% and three times in 100%, for 10 min each. Afterwards, they were dehydrated in tert-butoxide solution of 50%, 70%, 80%, 90% and three times in 100%, for 10 min each. Finally they were dried in a freeze-dryer for a period of 10 min^23^. The specimens were then examined with a TEM-1230 (JEOL, Japan).

### Physiological profiling using the Biolog EcoPlate approach

The Biolog EcoPlate approach was used to observe the utilization of different carbon and nitrogen sources by yeasts^24^. Single colonies were picked and streaked on the identification board using a wooden inoculum rod, followed by culture at 28 °C for 3 d according to the Biolog manual. Different color changes revealed the utilization of carbon and nitrogen sources by the strains. The results were analyzed and recorded using Biolog software (Microlog 34.20.05).

### Green synthesis of BioAgNPs

It was assumed that reducing substances produced by the yeasts take part in reducing Ag^+^ to BioAgNPs^25^, a silver nitrate solution was added to the yeast medium to a final concentration of 10 mM (Fig. 1 Step 2). First, the yeasts were seeded into the prepared medium, and cultured in a conical flask on a shaker at 28 °C and 200 rpm for 3 d. Then, the fermentation broths were collected and centrifuged to obtain the supernatants. The last step, silver nitrate solutions were then added into the obtained fermentation broths, and adjusted to a final concentration of 10 mM in a conical flask wrapped with tinfoil^26^. The color changes of the solutions were observed after 4 d culture at 28 °C in the dark^27^. The optimal synthesis conditions of BioAgNPs were determined, including Ag^+^ concentration, pH and reaction time^28^.

### Characterization of submicroscopic structures of the BioAgNPs

The submicroscopic structures of BioAgNPs was observed and analyzed by scanning electron microscopy (SEM) (Fig. 1 Step 3)^29^. The morphologies and microstructures of samples were observed by scanning electron microscopy (SEM, Hitachi Se4800). The SEM images were acquired at 10,000 × magnification.

### Antibacterial activity assay

Antibacterial activity assays were performed in order to determine the biological characteristics of BioAgNPs. The Kirby-Bauer (K-B) method was used to examine the inhibition of five pathogenic indicator strains by the BioAgNPs (Fig. 1 Step 4-I)^30^. Semi-solid media containing the pathogenic bacteria were poured into petri dishes. After solidification, filter papers containing yeast extracts were dripped onto the media, while 100 μg/mL ampicillin and 100 μg/mL kanamycin were utilized as positive controls, and PDA medium as negative control. After 24 h of culture at 26 °C, the diameters of the inhibition zones formed around the filter papers were measured, and the antibacterial effects of yeast extract were preliminarily demonstrated. Three biological replicates were included in each group, and the mean values were calculated and recorded^31^. The activity of AgNPs against the indicator strains was determined using standard minimum inhibitory concentrations (MIC) by measuring the zone of inhibition (ZOI)^32^.

### Cytotoxicity assay

MTT was used for cytotoxicity tests in order to characterize the biological properties of BioAgNPs (Fig. 1 Step 4-II)^33^. The A549 human lung cancer cell line^34^, H1975 and Beas 2B normal human lung epithelial cells were cultured and collected after enzymatic digestion. The obtained cells were then centrifuged at 200 g for 3 min, resuspended in culture medium, and adjusted to 100,000 cells/mL. The cells were then dispersed into wells of the 96-well plates and incubated for 24 h at 5% CO_2_, 37 °C and > 90% humidity.

Different amounts of BioAgNP solutions (0.1 μL, 0.25 μL, 0.5 μL, 1.0 μL, and 2.0 μL) were added into the media containing A549, H1975 and Beas 2B cells. Additionally, the same amount of sterilized distilled water was added in the control group. The plates were then incubated under the same condition for 24 h^35^.

After incubations, the media and BioAgNPs solutions were discarded, and 100 μL isopropanol was added to each well. The absorbance of the solutions at 490 nm was then measured using a microplate reader to calculate the relative viability of the cells.

### Statistics method

Excel 2016 (Microsoft Office Home & Student 2016) software was used to record and calculate the experimental data. Fitting analysis was carried out on the data of the parallel group. Data points that deviated from the fitting curve were discarded. The weighted average of the results of the other parallel groups was used to calculate the diameter of the inhibitory zone and assess cell viability.

## Results and discussion

### Identification of the yeast strains

**Figure 2:**
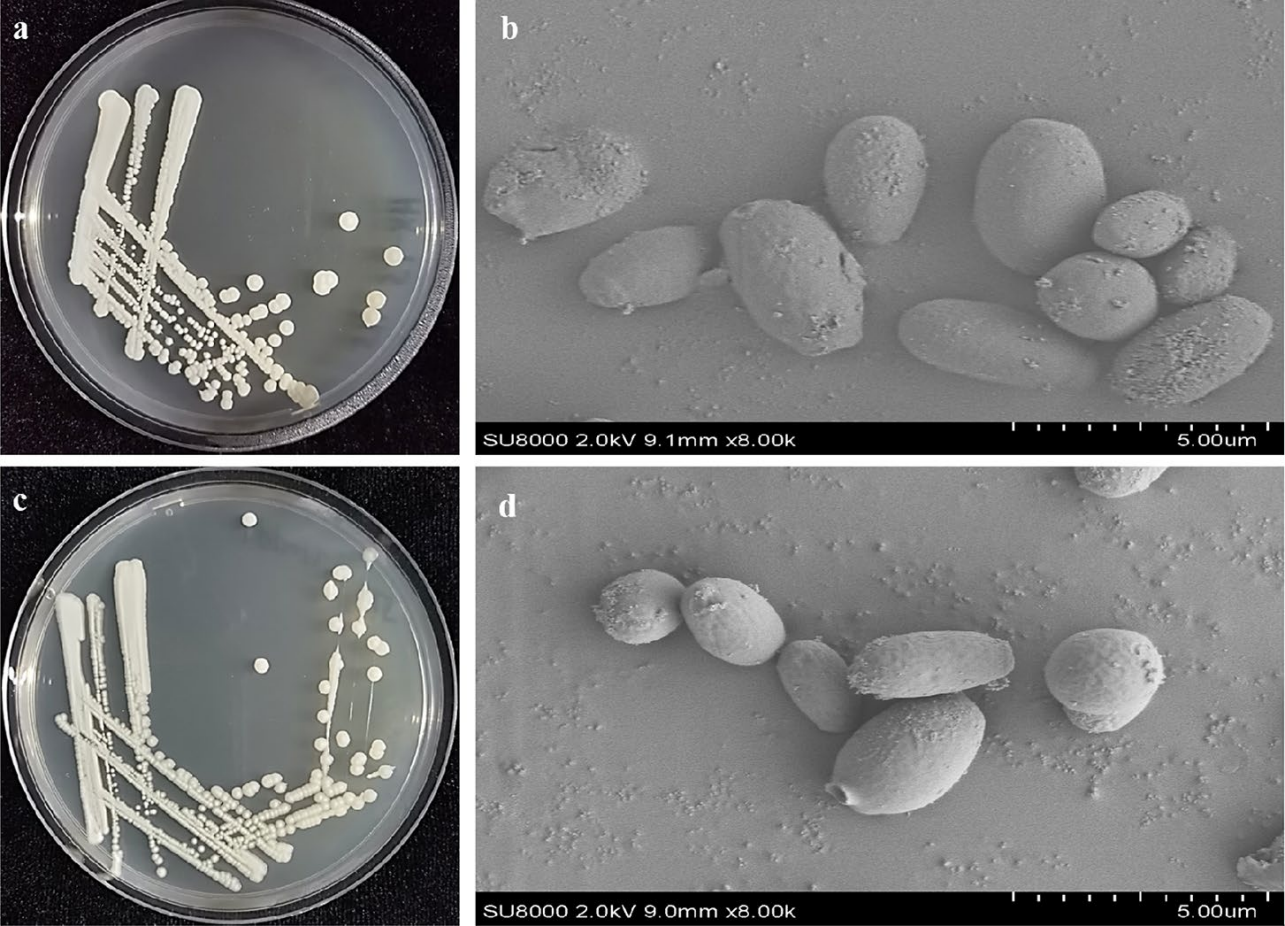
Colony morphology [(**a**) HX-YS; (**c**) LPP-12 Y] and SEM micrographs [(**b**) HX- YS; (**d**) LPP-12Y] (SEM at 2.0 kV and *8000).

The two yeast strains HX-YS and LPP-12Y were respectively isolated from surfaces of *Crataegus pinnatifida* and *Vitis vinifera* sampled in Qinghai province of China. Both strains had white round colonies with a powdery solid surface. During culture, the color of the colonies gradually changed from white to light brown, and finally became dark brown. The macroscopic colony appearance of both strains is shown in Fig. 2. The cells of both strains were elliptical, but those of HX-YS were slightly larger than those of LPP-12Y.

To determine the utilization of carbon and nitrogen sources of the two yeast strains HX-YS and LPP-12Y, the data were collected at 24 and 48 h (Table 1). The results showed that the utilization of carbon and nitrogen sources of the two yeasts was the same after 24 h of culture, but the utilization of L-Arginine and L-Serine was different after 48 h. Thus, no significant difference between HX-YS and LPP-12Y was observed in terms of substrate utilization.

**Table 1:** Utilization of 32 different carbon and nitrogen sources by HX-YS and LPP-12Y.

Nutrient substance	24 h		48 h	
	HX-YS	LPP-12Y	HX-YS	LPP-12Y
Water	—	—	—	—
β-Methyl-d-glucoside	++	++	++	++
d-Galactonic acid y-lactone	—	—	—	—
l-Arginine	++	++	++	+
Pyruvic acid methyl ester	—	—	—	—
d-Xylose	++	++	++	++
d-Galacturonic acid	—	—	—	—
l-Asparagine	—	—	—	—
Tween 40	+	+	+	+
I-Erythritol	—	—	—	—
2-Hydroxy benzoic acid	—	—	—	—
l-phenylalanine	—	—	—	—
Tween 80	+	+	+	+
d-Mannitol	++	++	++	++
4-Hydroxy benzoic acid	—	—	—	—
L-Serine	++	++	++	+
a-Cyclodextrin	—	—	—	—
N-Acetyl-d-glucosamine	++	++	++	++
y-Hydroxybutyric acid	—	—	—	—
l-Threonine	—	—	—	—
Glycogen	—	—	—	—
d-Glucosaminic acid	—	—	—	—
Itaconic acid	—	—	—	—
Glycyl-l-glutamic acid	—	—	—	—
d-Cellobiose	++	++	++	++
Glucose-1-phosphate	—	—	—	—
a-Ketobutyric acid	—	—	—	—
Phenylethyl-amine	—	—	—	—
a-d-Lactose	—	—	—	—
d,l-a-Glycerol	—	—	—	—
d-malic acid	—	—	—	—
Putrescine	—	—	—	—

“++”indicates that the strain can make full use of the nutrients of the pore plate; “+”indicates that the strain can't make full use of the nutrients of the pore plate; “—”indicates that the strain can't use the nutrients of this pore plate.

**Figure 3:**
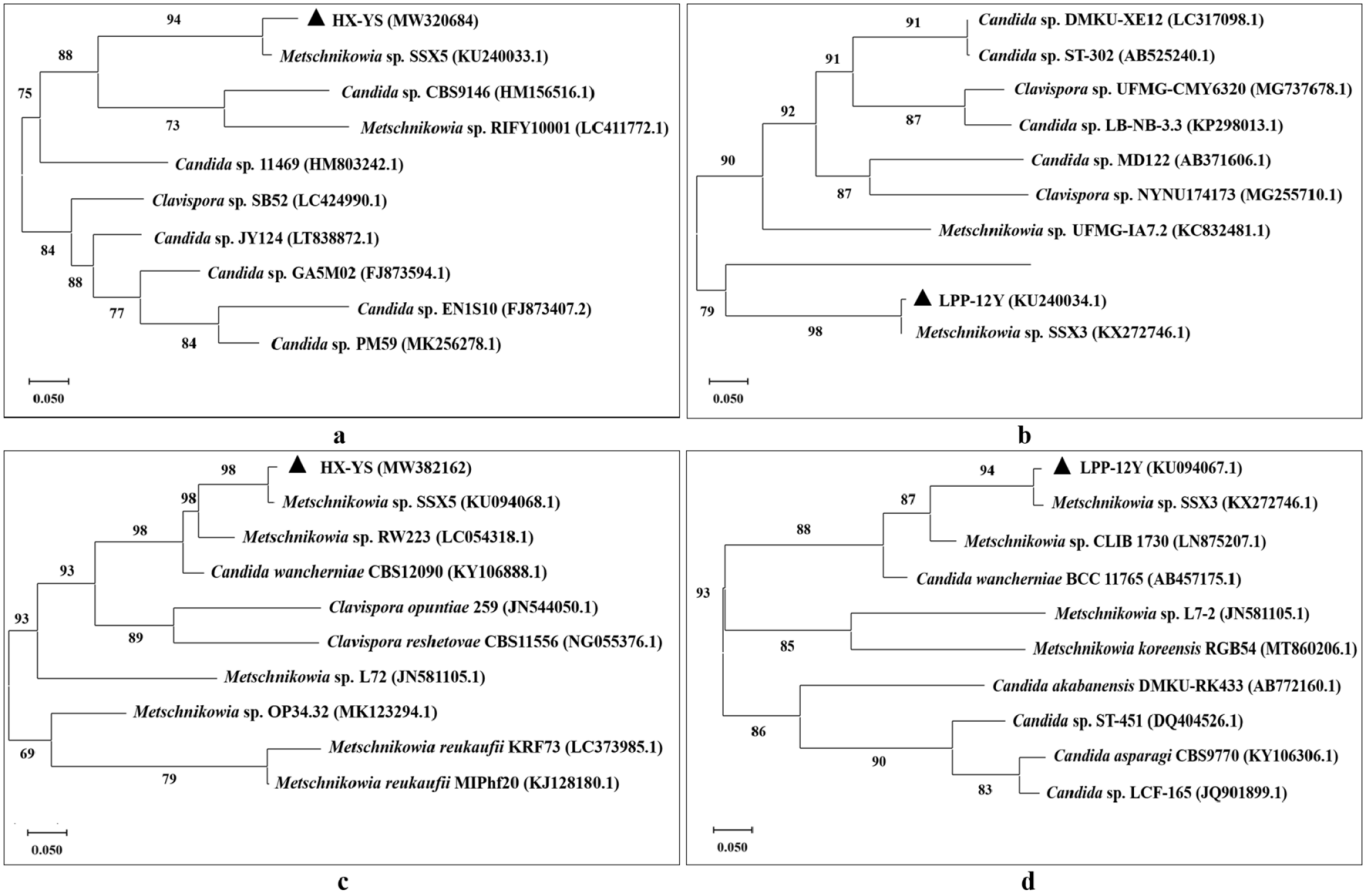
Phylogenetic tree based on the ribosomal DNA-ITS fragments [(**a**) HX-YS; (**b**) LPP-12Y] and 26S rDNA D1/D2 regions [(**c**) HX-YS; (**d**) LPP-12Y] of two novel yeasts strains constructed using the neighbor-joining method.

In order to further phylogenetically analyze HX-YS and LPP-12Y, the ribosomal DNA-ITS fragments and 26S rDNA D1/D2 regions were chosen for sequencing. The neighbor-joining trees the ITS sequences of HX-YS and LPP-12Y were shown in Fig. 3 a,b, and the trees of the 26S rDNA D1/D2 sequences of HX-YS and LPP-12Y were shown in Fig. 3 c,d. According to the ITS and 26S rDNA D1/D2 sequences, HX-YS, and LPP-12Y belongs to *Metschnikowia* sp. which showed 97% and 98% sequence similarity to HX-YS and LPP-12Y, respectively. The sequence similarity of HX-YS, and LPP-12Y with *Metschnikowia zizyphicola* was 98% and 96%, respectively. According to the morphological characteristics (Fig. 2), growth characteristics (Table 1), and the molecular analysis (Fig. 3)^36^, the HX-YS, and LPP-12Y strains were preliminarily identified as suspected novel species belonged to the genus *Metschnikowia*.

### Synthesis of BioAgNPs

**Figure 4:**
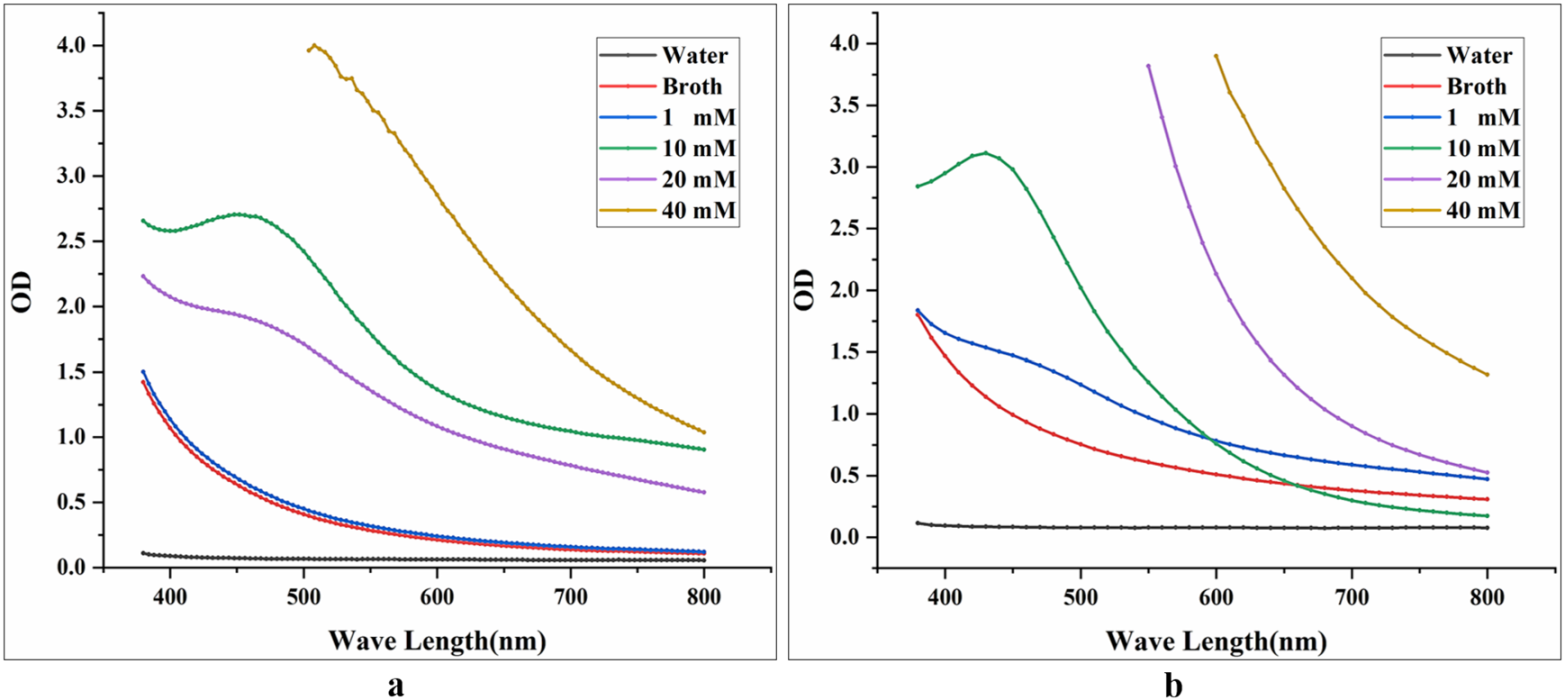
Absorption spectra of solutions with different concentrations of Ag^+^ (1 mM, 10 mM, 20 mM and 40 mM) ; water and broth of yeasts as control group, this reaction happens in a conical flask wrapped with tinfoil at 28 °C for 4 d [(**a**) solutions are treated with HX-YS; (**b**) solutions are treated with LPP-12].

Four concentrations of Ag^+^ (1 mM, 10 mM, 20 mM, 40 mM) were tested for the synthesis of BioAgNPs. It was found that 10 mM was the optimal Ag^+^ concentration for BioAgNP synthesis by both HX-YS and LPP-12Y (Fig. 4).

It can be seen from Fig. 4 that the maximum absorption peak of BioAgNPs synthesized by HX-YS appeared at the wavelength of 450 nm, compared to 430 nm for the particles synthesized by the yeast LPP-12Y. The absorbance values of the reaction system at wavelengths of 450 nm and 430 nm were measured, and the reaction concentration was then determined as 10 mM (Fig. 4). It can be observed that the synthesis of BioAgNPs began after the Ag^+^ and yeast fermentation broths were mixed. After 4 d, the absorbance gradually became stable. It could be inferred that the best time for synthesizing BioAgNPs by the two yeast was 4 d at an Ag^+^ concentration of 10 mM.

**Figure 5:**
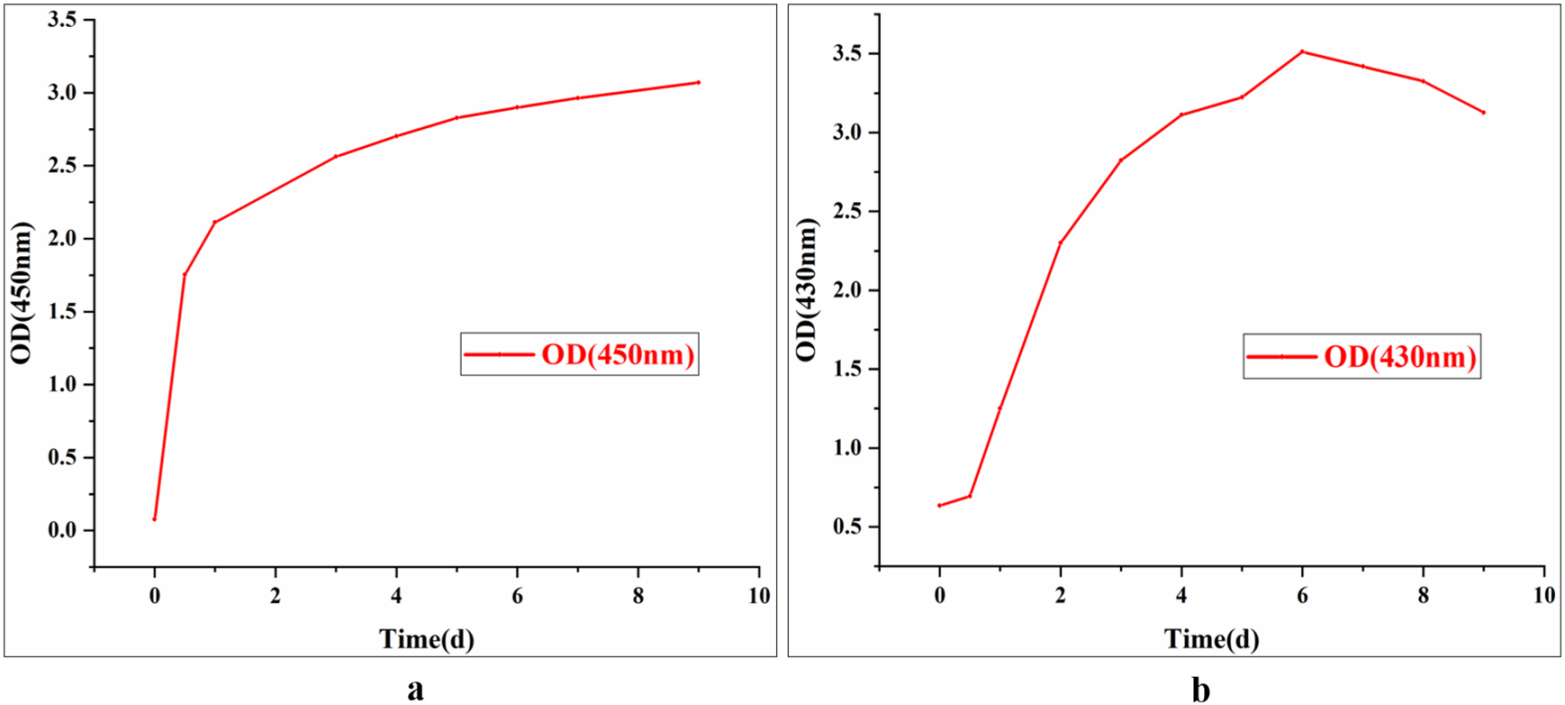
Absorption spectra of solutions with different pH (from 1.0 to 8.0) ; this reaction happens in a conical flask wrapped with tinfoil at 28 °C for 4 d [(**a**) solutions are treated with HX-YS; (**b**) solutions are treated with LPP-12].

The two yeast fermentation broths were reacted with Ag^+^ in the dark at different pH values. After 4d of reaction, the absorbance values of the reaction mixtures with different pH were measured, as shown in Fig. 5. The results indicated that the optimum pH for BioAgNP synthesis by the yeast HX-YS was 6.0, while for LPP-12Y it was 5.0. When exploring the optimal pH values for BioAgNP synthesis by the two yeasts, we found that the pH of the HX-YS broth needed to be increased from 4.0 to 6.0, meaning that the OH^−^ concentration of the reaction system increased, while the pH of the LPP-12Y broth needed to be reduced from 7.0 to 5.0. These results indicated that the functional relationship between the synthesis of BioAgNPs and the pH value is not a single linear relationship, as has been reported before^37^.

**Figure 6:**
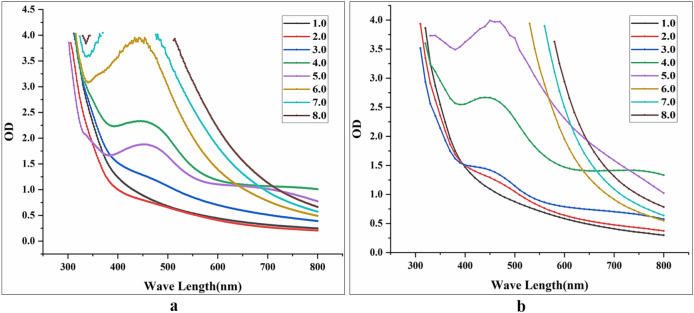
Optimal time for AgNPs synthesis: AgNPs solutions were synthesized under the optimal conditions at 28 °C in the dark, Ag^+^ concentration is 10 mM, and adjust the pH of HX-YS treated AgNPs solutions to 6.0, LPP-12Y is 5.0; OD value was determined everyday at the maximum absorption wavelength of AgNPs: the maximum absorption wavelength of AgNPs solutions treated with HX-YS fermentation broth is 450 nm, while LPP-12Y is 430 nm [(**a**) solutions are treated with HX-YS; (**b**) solutions are treated with LPP-12].

The content of BioAgNPs synthesized by HX-YS increased steadily during 10 d of synthesis, while synthesis by LPP-12Y began to decrease after 6 d. Moreover, the stability of BioAgNPs synthesized by the two yeast strains was different (Fig. 6). The BioAgNPs of HX-YS were more stable, but LPP-12Y was more efficient in the synthesis of BioAgNPs. Therefore, the optimal synthesis time for HX-YS was longer than 6 d, while LPP-12Y could complete the synthesis within the period of 6 d.

There are two theories about the biosynthesis mechanisms of biological nanomaterials, one postulating an enzymatic catalytic mechanism, and one predicting a nonenzymatic catalytic mechanism^38,39^. The enzymatic catalytic mechanism predicts that an enzyme acts as an electron transporter to transfer the electrons from reducing substances (such as reducing sugars) to the metal ions^38^. A possible non-enzymatic reduction mechanism may rely on quinine derivatives of naphthoquinones and anthraquinones produced by biological cells, which can also act as redox centers and do not require enzymes to reduce silver ions^39^. Regardless of the exact mechanism, it can be inferred that the synthesis of BioAgNPs is not only related to the concentration of OH^−^ and H^+^, but also closely related to the reducing substances in the yeast fermentation broth. Furthermore, the change of OH^−^ concentration will directly affect the reduction of Ag^+^, and also the activity of reducing substances in the yeast fermentation broth, which finally affects the synthesis of BioAgNPs.

If the synthesis mechanism of the yeast BioAgNPs can be determined, and the substances which play roles during fermentation could be identified, the conditions for the synthesis of BioAgNPs could be controlled exactly, which would further simplify the purification and application of BioAgNPs.

### Morphological characteristics of BioAgNPs

**Figure 7:**
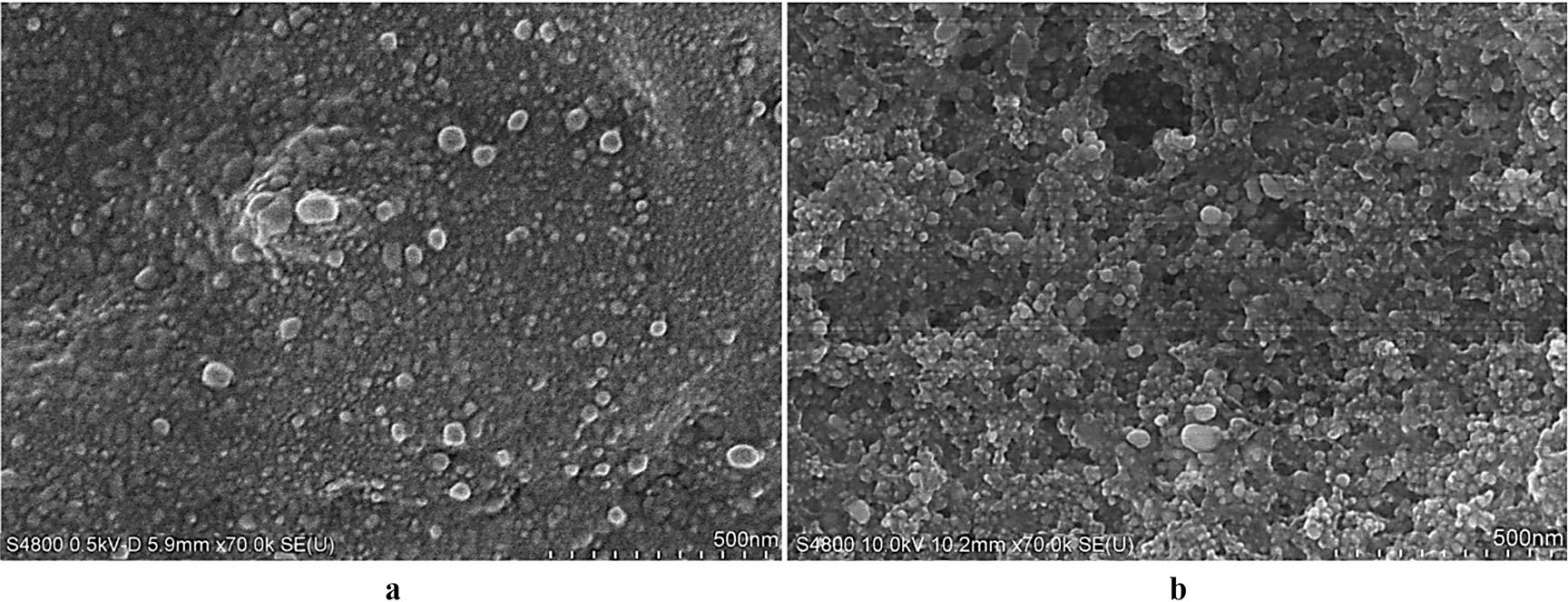
SEM images of AgNPs (AgNPs was synthesized under the optimal conditions at 28 °C in the dark for 6d, Ag^+^ concentration is 10 mM, and adjust the pH of HX-YS treated AgNPs solutions to 6.0, LPP-12Y is 5.0) [(**a**) AgNPs synthesized by HX-YS; (**b**) AgNPs synthesized by LPP-12].

The microstructure of the BioAgNPs synthesized by the yeast strains LPP-12Y and HX-YS was analyzed by scanning electron microscopy (SEM; Fig. 7 ). It can be seen from the figure that the BioAgNPs synthesized by the two yeasts were similar. They displayed a spherical morphology with sizes ranging between 50 and 100 nm. However, the BioAgNPs synthesized by HX-YS were significantly smaller and more uniform than those synthesized by LPP-12Y.

The microstructure of the BioAgNPs synthesized by the two yeasts was very similar to the BioAgNPs synthesized by *Neurospora intermedia* in a previous study^40^.

### Antibacterial activity of the BioAgNPs

**Figure 8:**
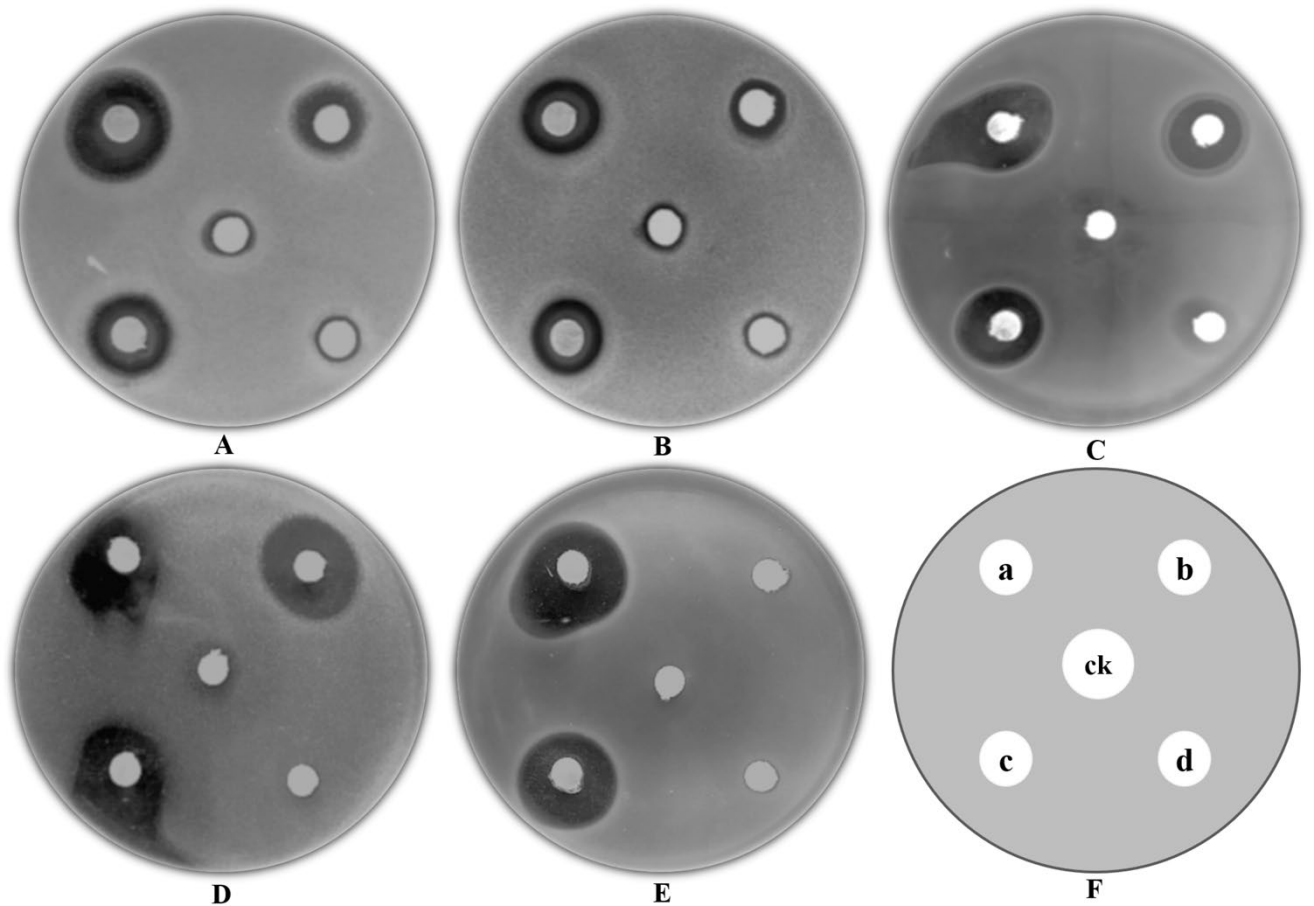
Antimicrobial activity of silver nanoparticles against various pathogenic bacterial strains shown by filtering paper diffusion method [(**A**) *E. coli* ATCC8099; (**B**) *S. aureus* ATCC6538; (**C**) *B. subtilis* ATCC6051; (**D**) *M. albican* ATCC10231; (**E**) *P. aeruginosa*)] (a: AgNPs from LPP-12Y; b: 100 μg/mL Kanamycin; c: 100 μg/mL Ampicillin; d: AgNPs from HX- YS; ck: PDA).

The antibacterial effects of BioAgNPs synthesized by LPP-12Y and HX-YS against five pathogenic bacteria, including *E. coli* ATCC8099, *S. aureus* ATCC6538, *B. subtilis* ATCC6051, *M. albicans* ATCC10231, and *P. aeruginosa*, was assessed by measuring the diameters of the inhibition zones^41^. As can be seen in Fig. 8, round filter papers were put on each plate, with different substances including fermentation broths of the two yeast strains, 100 μg/mL ampicillin(as positive controls), 100 μg/mL kanamycin (as positive controls) and PDA medium (as negative control). In the Kirby-Bauer method, if the diameter of the inhibition zone is less than 10 mm, it means that the tested substance has a low inhibitory effect on the pathogenic bacteria and it can be considered that the pathogenic bacteria are not sensitive to the tested substance (based on the 2004 NCCLS standard). The results showed that the BioAgNPs synthesized by the two yeast strains had obvious antibacterial effects against all 5 pathogenic bacteria (Table 2). The five pathogenic bacteria were generally not sensitive to the tested antibiotics, and only kanamycin showed an efficient inhibitory effect against *B. subtilis* ATCC6051. In addition, the LPP-12Y and HX-YS BioAgNPs showed the best antibacterial effect against *P. aeruginosa* with the largest inhibition zone reaching 22.29 mm. The antibacterial activity of the BioAgNPs against *E. coli* ATCC8099 and *S. aureus* ATCC6538 was 2 times higher than that of 100 μg/mL ampicillin, as well as 1.5 times that of 100 μg/mL kanamycin. Therefore, the AgNPs synthesized by HX-YS and LPP-12Y had good antibacterial effects. The BioAgNPs formed by LPP-12Y showed better inhibitory effects against *E. coli* ATCC8099, *S. aureus* ATCC6538, *B. subtilis* ATCC6051 and *P. aeruginosa*, than those formed by HX-YS, while the latter displayed a better inhibitory effect against *M. albicans* ATCC10231 than the former.

**Table 2:** Antimicrobial activity of AgNPs synthesized by HX-YS and LPP-12Y against five pathogenic microorganisms.

Pathogenic microorganism	Zone of Inhibition (mm)				
	*E. coil* ATCC8099	*S. aureus* ATCC6538	*B. subtilis* ATCC6051	*M. albican* ATCC10231	*P. aeruginosa*
PDA	–	–	–	–	–
Kanamycin	10.83 ± 0.57	10.43 ± 1.55	15.05 ± 1.51	19.22 ± 0.31	–
Ampicillin	7.51 ± 0.10	7.92 ± 0.54	11.44 ± 0.80	–	–
Sample HX-YS	14.81 ± 1.31	14.61 ± 1.72	15.29 ± 0.88	18.52 ± 0.45	21.44 ± 1.49
Sample LPP-12Y	15.23 ± 4.08	15.04 ± 1.31	16.23 ± 1.39	17.83 ± 1.22	21.78 ± 0.51

* The zone of inhibition (ZOI) is recorded as the mean ± SD (n = 3). The diameter of the disks is 6 mm. “–” means no ZOI was observed.

Silver generally acts as a disinfectant, and when the size of elemental silver particles reaches the nanometer scale, the antibacterial effect of the silver can be greatly enhanced^42^. In this study, the two yeast strains efficiently synthesized BioAgNPs, which displayed good antibacterial activity, significantly better than 100 μg/mL ampicillin and 100 μg/mL kanamycin. Thus, the BioAgNPs synthesized by the yeast strains LPP-12Y and HX-YS should be explored for the further development and application of BioAgNPs technology.

The size, surface area and morphology of silver nanoparticles affect the biological activity of silver nanoparticles^43,44^. The bactericidal properties of silver nanoparticles with different structures are different, mainly because the crystal surfaces of silver nanoparticles with different structures are not consistent. Cubic silver nanoparticles have more reactive crystal surfaces and better bactericidal effect, while spherical silver nanoparticles have relatively stable exposed crystal surfaces, so the bactericidal effect is relatively poor^45^. Barabadi further analyzed the bacteriostatic mechanism of nano-silver and found that reactive oxygen species would be generated in the contact process between nano-silver and bacteria, and the oxidation reaction induced by these reactive oxygen species was the main bactericidal mechanism of nano-silver. Therefore, it is preliminarily speculated that the antimicrobial activity of silver nanoparticles with different structures and crystal faces is significantly different, mainly because their structure and crystal faces determine their ability to produce reactive oxygen species, thus determining their biological activity.

### Antitumor effects and biocompatibility of BioAgNPs

**Figure 9:**
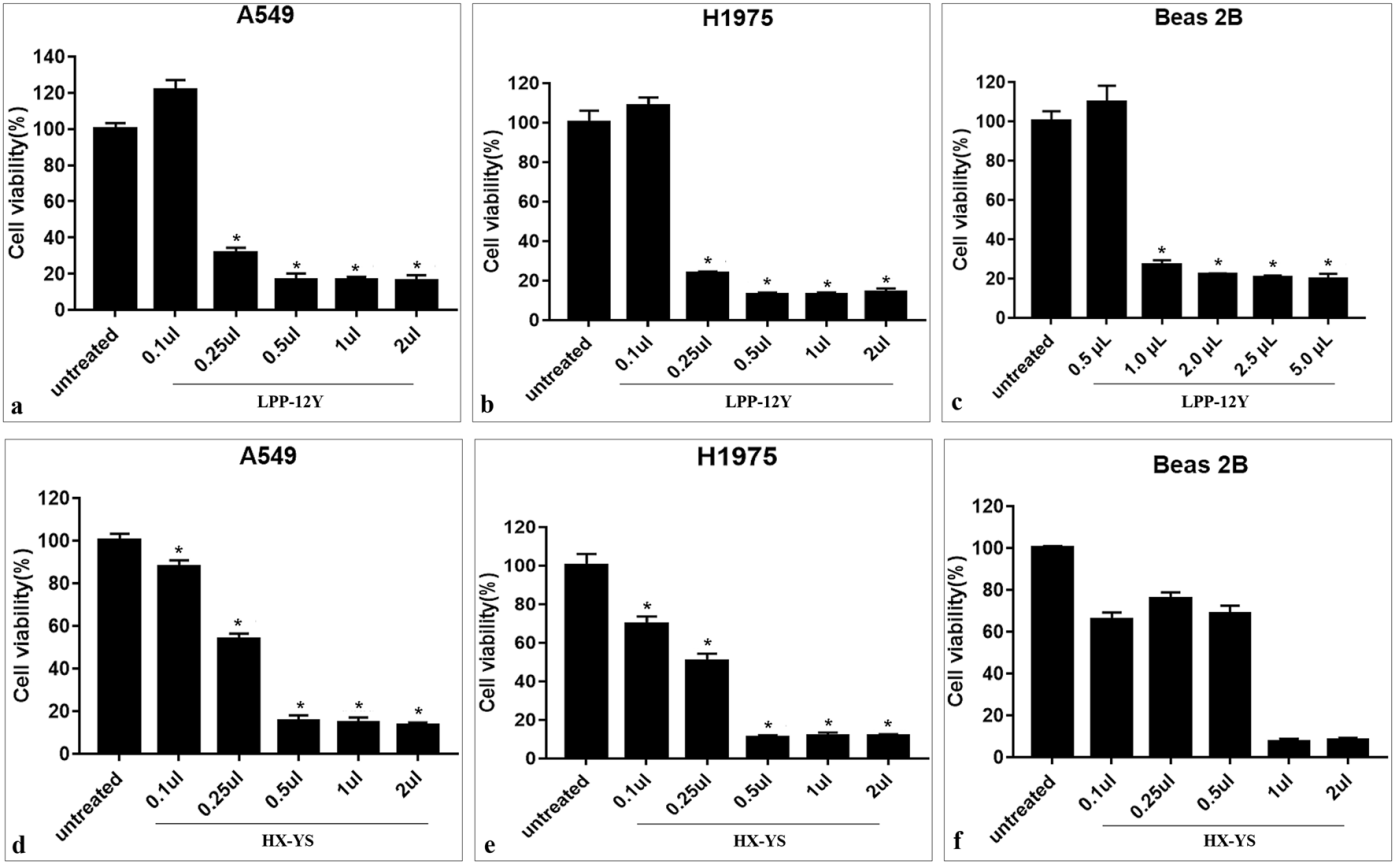
Histogram showing cellular viability of A549 and H1975 and Beas 2B cells after exposure to different concentrations of AgNPs. The untreated cells by AgNPs as control is included.

The antitumor effects of the BioAgNPs formed by HX-YS and LPP-12Y were assessed using human lung cancer cell lines A549 and H1975, and their biocompatibility was detected assessed with Beas 2B normal human lung epithelial cells. The cells were cultured with the BioAgNPs, after which the viability and morphology of cells was assessed. At a dose of 0.5μL, the BioAgNPs showed a significant inhibitory effects against A549 and H1975 cells, with a significant decrease of cell viability to below 20%. Moreover, there was only a much weaker inhibitory effects against the Beas 2B normal human lung epithelial cells (Fig. 9).

Therefore, it can be inferred that BioAgNPs formed by LPP-12Y had a specific inhibitory effect on cancer cells with a much weaker inhibitory effect on normal cells at about 0.25 μl/mL, and the BioAgNPs produced by HX-YS also had a considerable effect and tumor specificity, but at a higher concentration than the BioAgNPs produced by LPP-12Y. Therefore, the BioAgNPs formed by the yeast strains LPP-12Y and HX-YS may have potential for cancer treatment.

With the development of modern medicine and health technologies, the application of nanomaterials has become more widespread, and various biological effects of nanomaterials have been reported. The results showed that the transplanted material did not induce any pathological phenomena, indicating the good biocompatibility of AgNPs. The biocompatibility of AgNPs in hygiene products was also assessed through t in-vivo vaginal irritation and intracutaneous reactivity studies. The results showed that when 0.2−0.4 g of AgNPs were added per hygiene product, the irritation caused by the addition of AgNPs could be completely eliminated while maintaining strong antibacterial activity^46^. In a further study, AgNPs were synthesized using extracts of tobacco stems, which not only had no toxicity to the nerve cells of mice, but also had a certain protective effect.

In this study, the BioAgNPs synthesized by the yeast strains HX-YS and LPP-12Y displayed a low toxic effect on normal cells, but showed an effective inhibitory capability against lung cancer cell lines A549 and H1975. This result suggests that the yeast HX-YS as well as the BioAgNPs synthesized by it merit further investigation, with special attention to possible medical applications.

## Conclusions and outlook

Based on the ITS rDNA genes, 26S rDNA D1/D2 regions and relevant characteristics, the yeast strains HX-YS and LPP-12Y isolated from the surfaces of *C. pinnatifida* and *V. vinifera* respectively in Qinghai province of China were identified as belonging to the genus *Metschnikowia*. The fermentation broth of the strains HX-YS and LPP-12Y was used to synthesize BioAgNPs which showed various notable biophysical properties. The optimal synthesis conditions of BioAgNPs with the strain HX-YS were found to be 10 mM Ag^+^, pH 6 and culture duration of more than 6 d, while the conditions for strain LPP-12Y were 10 mM Ag^+^, pH 5, and 6 d. The BioAgNPs synthesized by the two yeast strains showed strong antibacterial effects, inhibited the growth of cancer cells and had good biocompatibility with normal cells. Therefore, the yeasts LPP-12Y and HX-YS, which are suspected novel species of the genus *Metschnikowia*, and have new biosynthesis ability of bioAgNPs as well as their BioAgNPs have the potential to contribute to the development and application of nanomaterials.

In this study, the biosynthesis of silver nanoparticles was investigated, and the antibacterial activity and anti-cancer effect of silver nanoparticles were preliminarily analyzed. However, this paper still has some deficiencies. The experiment has not analyzed in detail the specific substances that play a role during the process of silver nanoparticle biosynthesis in yeast. The antibacterial effect and anti-cancer effects of nano-silver were preliminarily analyzed, but more details of the antibacterial effect and anti-cancer mechanism of nano-silver were not included. Therefore, future studies should elucidate the synthesis mechanism of nano-silver, factors influencing the morphology of nano-silver, as well as the mechanism of antimicrobial and anticancer activity.
